# BDKD-Net: Boundary-Probability Knowledge Distillation for Compact Polyp Segmentation

**DOI:** 10.3390/jimaging12070306

**Published:** 2026-07-08

**Authors:** Tian Xia, Jianhua Li, Liping Sun

**Affiliations:** College of Medical Instruments, Shanghai University of Medicine & Health Sciences, Shanghai 201318, China; 243352316@st.usst.edu.cn (T.X.); lijh@sumhs.edu.cn (J.L.)

**Keywords:** polyp segmentation, knowledge distillation, boundary supervision, compact deep learning, endoscopic imaging, medical image analysis

## Abstract

Accurate polyp segmentation in colonoscopy supports early detection of colorectal cancer, but compact models under a student-only inference budget tend to lose boundary fidelity. BDKD-Net is a 3.72M-parameter compact student trained under a composite knowledge-distillation loss that combines a response signal, a boundary-probability signal restricted to a static teacher-derived boundary band, and an auxiliary detail-feature alignment; the teacher is used only during training and discarded at inference. In a uniform five-seed re-run on a locked Kvasir-SEG and CVC-ClinicDB development split, the same-architecture scratch student reaches Dev Dice 0.9210 ± 0.0029 and boundary F1 at 3-pixel tolerance 0.7726 ± 0.0057, while full BDKD-Net reaches 0.9300 ± 0.0029 and 0.8008 ± 0.0094. Boundary-probability distillation is the load-bearing signal: it has the highest mean Dev ${BF1}_{t3}$ among the single KD signals and carries the boundary gain at no inference cost, with the full model reaching four-external Dice 0.8225 ± 0.0071 at 1.76 GFLOPs and 140.9 FPS. Among the directly reproduced baselines, the higher-Dice Polyp-PVT (0.8417 ± 0.0091) needs 6.8× the parameters and 5.7× the compute at roughly half the frame rate. BDKD-Net thus delivers a compact, boundary-faithful student that keeps most of the accuracy of much larger models at a fraction of their inference cost.

## 1. Introduction

Compact polyp segmentation must hold three properties at the same time—accurate region overlap, faithful polyp boundary localization, and low student-only inference cost—and the standard route of shrinking a full-size encoder typically gives up boundary fidelity before it gives up region overlap. Colonoscopic polyps frequently appear with low contrast against the surrounding mucosa and with diffuse rims, so a segmentation that is locally accurate inside the lesion but smeared at the rim is weaker for downstream review than its region score alone would suggest [1,2,3]. At the same time, compact inference targets cap parameter count, floating-point operations, and per-frame latency well below those of full-size segmentation backbones [4,5]. The pressure point for a compact model is, therefore, boundary preservation under a fixed inference budget, since the region-overlap target can be reached without dedicated boundary supervision, but the boundary itself is what compact backbones lose first.

Architecture-side polyp segmentation has invested heavily in boundary-aware decoders, attention and context modules, and multi-scale fusion to recover the boundary signal that pure encoder shrinkage tends to erode, yet these routes still rely on enough inference-time capacity for the auxiliary branches, which is what the compact-student budget removes. On the backbone side, reverse-attention decoders, pyramid Transformer backbones, and cross-level fusion improve foreground localization, with their auxiliary boundary branches assuming that the encoder can absorb the added gradient [6,7,8,9]. Methods that embed boundary attention or edge guidance inside the main segmentation network couple boundary fidelity to inference-time capacity by keeping the boundary branch at run time [10,11,12]. Lightweight polyp architectures reach low parameter counts through context-aware modules and linear-complexity attention, yet in published evaluations on the standard polyp benchmarks they still trail full-size models on region-overlap and boundary-sensitive scores [4,5,13]. Boundary supervision is, therefore, a well-developed architectural component, and what the compact-student setting still lacks is a channel through which it reaches the student without inheriting the inference-time capacity these routes require.

Knowledge distillation offers the candidate channel for this role because a high-capacity teacher can supply supervision during training while the compact student carries the inference cost on its own [14]. The foundational route transfers softened output probabilities from teacher to student [15], and a complementary route additionally matches intermediate feature representations between teacher and student [16]; BDKD-Net’s response and detail-feature signals draw on this response-level and feature-level lineage, respectively, restricted here to a compact polyp-segmentation student. Three concrete polyp-segmentation routes have already explored this idea. KDAS uses attention-supervision distillation: it transfers the teacher’s attention maps to a compact polyp student and treats teacher–student attention inconsistency as the distillation signal [17]. PRISM uses self-distillation: it constructs the teacher as the exponential moving average of the student’s own weights, so the supervision signal is a temporally smoothed student prediction [18]. MicroAUNet (preprint) uses two-stage progressive distillation: it transfers semantic and boundary cues to a compact student in two progressive stages rather than as a single combined objective [19]. A related edge-guided, feature-aligned KD route has also been reported [20]. Recent lightweight or boundary-focused polyp methods also include EMCAD [21], LGPS [22], DepthPolyp [23], LiteBounD [24], De-LightSAM [25], and MEGANet-W [26]; where runnable artifacts are available, we evaluate them under the same external protocol, and otherwise cite them only as method references. Across these routes, the supervision signal is attention-level, EMA-prediction-level, progressively organized semantic-then-boundary, edge/feature-aligned, pseudo-depth-guided, or foundation-model-guided; what they leave open as a signal-design question is explicit boundary-probability supervision on the teacher boundary band.

We propose BDKD-Net, a 3.72M-parameter compact polyp segmentation student trained under the composite objective *L* = $L_{\mathrm{seg}}$ + 0.5·$L_{\mathrm{resp}}$ + 1.0·$L_{\mathrm{bd}}$ + 0.25·$L_{\mathrm{detail}}$, where a PVTv2-B2 [27] + EGFR teacher is used only during training. The main contribution is boundary-probability knowledge distillation on a static teacher-derived boundary band: restricting the binary cross-entropy between student and teacher probability to the morphology-dilated band of the teacher mask has the highest mean Dev ${BF1}_{t3}$ among the single KD signals, reaching Dev ${BF1}_{t3}$ 0.7990 ± 0.0127 compared with 0.7726 ± 0.0057 for the scratch student and 0.7860 ± 0.0062 for response distillation alone. The auxiliary detail-feature alignment is a context-dependent supporting signal: alone, it is close to scratch, but within the full combination it contributes to the strongest four-external Dice in the same-architecture KD ablation family. We position BDKD-Net as a compact-student efficiency–accuracy operating point.

## 2. Methods

**Figure 1:**
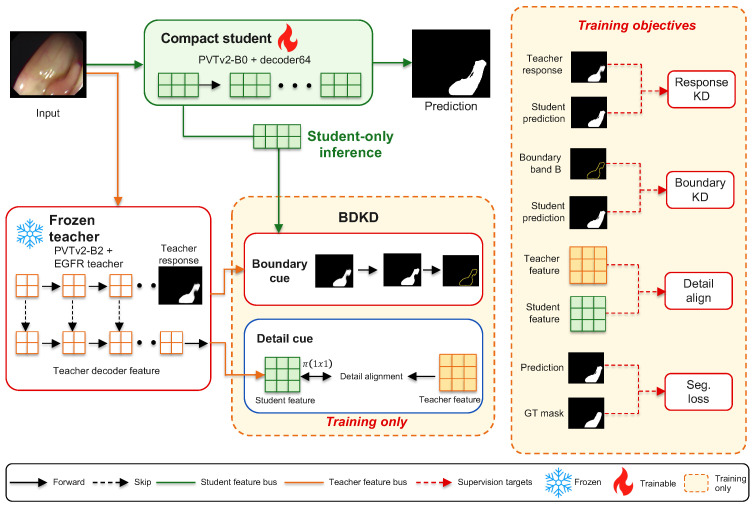
BDKD-Net training-time pipeline. A frozen PVTv2-B2 + EGFR teacher (left) supervises a compact PVTv2-B0 + decoder64 student through three training-time distillation signals: response (teacher prediction → student prediction), boundary-band probability (boundary band *B* derived from the morphology-dilated 0.5-thresholded teacher mask → student prediction), and detail feature (teacher decoder feature → student decoder feature). The four training objectives appear on the right. Inference is student-only; the teacher is discarded after training.

Specifically, BDKD-Net is a teacher–student training graph (Figure 1) in which a 26.06M PVTv2-B2 [27] + EGFR teacher supervises a 3.72M compact polyp student through three distillation signals at training time, and the inference graph is the 3.72M student alone. Here, EGFR denotes an Edge–Gated Frequency-Residual decoder head (unrelated to the epidermal growth factor receptor): a Sobel edge-enhancement branch and a frequency-domain residual-enhancement branch are combined through a learned sigmoid gate before the segmentation head. The teacher pairs a PVTv2-B2 backbone with this EGFR-gated edge head, loads from a frozen checkpoint, and stays in evaluation mode for every iteration. The student pairs a PVTv2-B0 [27] backbone with a decoder of width 64 and a single 1  ×  1 segmentation head, and a downstream detail adapter inside the decoder pathway carries the auxiliary detail-feature alignment; the adapter operator is defined below. The three distillation signals—response, static teacher-derived boundary-band probability, and detail feature—couple the student’s decoder features and probability map to the teacher’s, so boundary supervision reaches the student through the loss rather than through a runtime branch, and the inference graph is the 3.72M student alone. The following paragraphs define, in order, the teacher provenance, data, and split; the loss formulation; the student backbone, detail adapter, and training schedule and the evaluation protocol.

The training data and the external evaluation data come from disjoint pools: Kvasir-SEG [2] and CVC-ClinicDB [28] are pooled for student training and internal validation, while four external polyp-segmentation benchmarks are reserved for evaluation and are not used for tuning or model selection. The teacher recipe used for training is M2_boundary_self_distill, a PVTv2-B2 + EGFR E_gated_F segmentation network (26.06M parameters) obtained by self-distillation on the in-domain Kvasir-SEG + CVC-ClinicDB pool; this recipe was fixed a priori and was not selected from the sensitivity comparison described below. Within this fixed recipe, the training checkpoint is selected by best Dev Dice across three seeds (0.9552, 0.9496, 0.9473), loaded once, frozen, kept in evaluation mode, and used only to supply the response, static boundary-band probability, and detail-feature targets to the student during training; no external dataset is used for teacher training, checkpoint selection, student training, or student checkpoint selection. To characterize whether the choice of teacher recipe is a fragile, cherry-picked decision, four alternative teacher recipes were separately profiled on the same fixed development split, each evaluated across five seeds: Base 0.9438 ± 0.0046, M2 0.9475 ± 0.0061, M2_gated_edge_boundary 0.9494 ± 0.0023, and M6_efficient 0.9502 ± 0.0030 Dev Dice. The recipe used sits within this 0.944–0.950 band, and no single recipe in the comparison dominates the others by a margin that exceeds their own seed variance, so the teacher recipe is not an outlier choice within this local comparison. This reports seed-variance across candidate teachers on one pre-registered fixed development split, not k-fold cross-validation on disjoint folds, and the five-seed recipe comparison and the three-seed checkpoint selection are two distinct procedures rather than one combined selection rule; formal k-fold validation of teacher selection is left as future work. The internal pool combines all Kvasir-SEG and CVC-ClinicDB image–mask pairs, shuffles them with a fixed random seed of 42, and reserves 10% as the internal-validation split, yielding 1451 training images and 161 internal-validation images.

External evaluation uses CVC-ColonDB [29] (380 images), CVC-300 [3] (60), ETIS-LaribPolypDB [30] (196), and BKAI-IGH-NeoPolyp (the first 512 image–mask pairs in ascending lexicographic filename order from a 1000-image source pool; no random or manual selection was applied); predictions are binarized with one fixed sigmoid threshold of 0.5, CVC-ColonDB, CVC-300, and ETIS masks are binarized at intensity 127, and BKAI masks are binarized at 0 because its public masks use a positive-value encoding. All images are resized to 352 × 352 and normalized with ImageNet statistics; training augmentation applies random flips, 90° rotations, color jitter, and Gaussian blur, and evaluation applies resize and normalization only.

**Figure 2:**
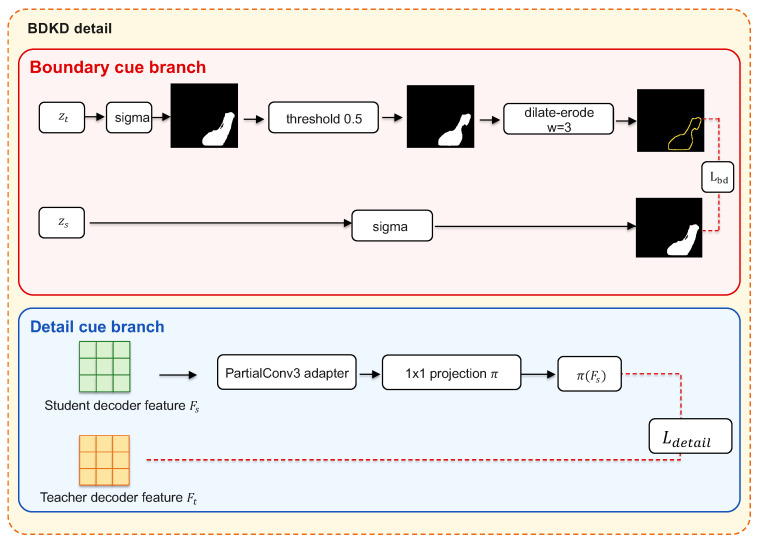
Construction of the boundary cue and detail cue inside the BDKD interface. (**Top**) the boundary band *B* is built by passing the teacher logit $z_{t}$ through sigma, thresholding at 0.5 to obtain the teacher mask $M_{t}$, and applying a morphological dilate-erode with width 3; restricting the binary cross-entropy between $\sigma (z_{s})$ and $\sigma (z_{t})$ to band *B* yields $L_{\mathrm{bd}}$. (**Bottom**)the student decoder feature $F_{s}$ passes through a PartialConv3 adapter and a 1×1 projection π, and a squared L2 against the teacher decoder feature $F_{t}$ forms $L_{\mathrm{detail}}$.

The student is trained against a composite objective in which the supervised segmentation term, the response distillation term, the boundary-band probability distillation term (Figure 2), and the detail-feature alignment term play distinct roles:

(1) $$L=L_{\mathrm{seg}}+0.5L_{\mathrm{resp}}+1.0L_{\mathrm{bd}}+0.25L_{\mathrm{detail}}.$$

The supervised segmentation term combines binary cross-entropy on logits with Dice loss on probabilities:

(2) $$L_{\mathrm{seg}}=L_{\mathrm{BCE}}^{\mathrm{logit}}\left(z_{s},y\right)+L_{\mathrm{Dice}}\left(\sigma \left(z_{s}\right),y\right).$$

The response distillation term also uses the logit-form binary cross-entropy, with the teacher probability map as the soft target:

(3) $$L_{\mathrm{resp}}=L_{\mathrm{BCE}}^{\mathrm{logit}}\left(z_{s},\sigma \left(z_{t}\right)\right).$$

The teacher hard mask is obtained from the teacher probability map using a fixed threshold:

(4) $$M_{t}=1\left[\sigma \left(z_{t}\right)>\theta \right],\;\theta =0.5.$$

The boundary band is then constructed by morphological dilation and erosion with the fixed band width used in the main protocol:

(5) $$B=\mathrm{dilate}\left(M_{t};w\right)-\mathrm{erode}\left(M_{t};w\right),\;w=3.$$

The boundary-band probability distillation term restricts probability-space binary cross-entropy to this teacher-derived band:

(6) $$L_{\mathrm{bd}}=\frac{\sum_{i}B_{i}L_{\mathrm{BCE}}^{\mathrm{prob}}\left(\sigma \left(z_{s,i}\right),\sigma \left(z_{t,i}\right)\right)}{\sum_{i}B_{i}+\epsilon }.$$

For the detail-feature term, the student decoder feature is first projected to the teacher feature channel space:

(7) $${\tilde{f}}_{s}=\pi \left(f_{s}\right).$$

The detail alignment loss is then a squared feature error:

(8) $$L_{\mathrm{detail}}={∥{\tilde{f}}_{s}-f_{t}∥}_{2}^{2}.$$

Here $L_{\mathrm{BCE}}^{\mathrm{logit}}$ denotes BCE-with-logits, while $L_{\mathrm{BCE}}^{\mathrm{prob}}$ denotes ordinary BCE on probabilities. The only spatial restriction in the implemented loss is the teacher-derived boundary band in Equations (4)–(6). The component weights 0.5, 1.0, and 0.25 are taken from the executed training-code path that produced the main full-confirm run; an earlier pilot configuration that listed a detail-feature weight of 0.5 was not used for final training and is retained only as a deprecated supplementary record.

The student is a compact PVTv2-B0 backbone followed by a decoder of width 64 and a single 1 × 1 segmentation head, with a PartialConv3 [31] detail adapter inside the decoder pathway that carries the detail-feature alignment signal defined in Equation (8). The PartialConv3 adapter applies a depthwise 3 × 3 convolution to a subset of channels and then mixes the result with the remaining channels through a 1 × 1 projection that returns the full channel space. The a priori rationale for this design is efficiency: restricting the spatially-aware 3 × 3 kernel to a channel subset, rather than convolving the full channel width as a standard 3 × 3 convolution does, lowers the multiply-add count of the adapter while the subsequent 1 × 1 projection still mixes information across all channels before the aligned feature is compared against the teacher. PartialConv3 is used here as a design choice inside the detail-feature alignment pathway and is not framed as a standalone architectural innovation; the trade-off between PartialConv3 and a standard 3 × 3 convolution at the same channel width is quantified empirically in the Results section, where PartialConv3 reaches lower GFLOPs and a paired-seed advantage in external generalization over the standard-convolution variant, substantiating the a priori efficiency rationale with the observed accuracy–efficiency trade-off. Every student variant is trained under the same recipe: AdamW [32] with learning rate 1 × 10^−4^ and weight decay 1 × 10^−4^; a cosine [33] learning-rate schedule; a 30-epoch upper bound with patience-7 early stopping on internal-validation Dice; batch size 8, degraded to 4 or 2 when GPU memory is tight; automatic mixed precision and the teacher branch frozen and in evaluation mode for every iteration. The checkpoint selected for evaluation is the one with the best internal-validation Dice, a Dev-side selection metric chosen because the boundary endpoint is unstable in early epochs and Dev Dice provides a more reliable convergence anchor. The locked-split ${BF1}_{t3}$ endpoint reported in Section 3 is computed only on this selected checkpoint, holding the fairness anchor identical across the student control group and the BDKD-Net variants and keeping the selection metric distinct from the headline endpoint.

Every student variant is re-trained under five random seeds (42, 1888, 2024, 9999, 31,415), and the locked internal dev split is evaluated under two metrics that target region overlap and boundary fidelity separately. Region overlap is reported as internal-validation Dice, and boundary fidelity is reported as boundary F1 at a 3-pixel tolerance, taken as the main boundary endpoint of this paper. As a sensitivity check, boundary F1 is also recounted at 1- and 5-pixel tolerances without changing the selected checkpoint. External evaluation is reported as a four-set average over CVC-ColonDB, CVC-300, ETIS-LaribPolypDB, and BKAI-IGH-NeoPolyp. Efficiency is measured on a single RTX 4090 under automatic mixed precision at input size (1, 3, 352, 352), with 30 warmup iterations and 100 timed iterations and GFLOPs counted by thop [34]; only the student is profiled at inference, and the values reported under this protocol are not directly comparable to literature numbers measured under different hardware or settings.

Claude Code (Anthropic; Claude Opus 4.7 and 4.8) was used during the preparation of this manuscript only, as detailed in the back-matter declaration; it was not used to generate, alter, or infer any experimental data, result values, statistical outputs, or figure images.

## 3. Results

On the locked Kvasir-SEG [2] + CVC-ClinicDB [28] internal dev split, the 3.72M-parameter compact BDKD-Net student trained under the full A+B+C combination reaches Dev Dice 0.9300 ± 0.0029 and Dev ${BF1}_{t3}$ 0.8008 ± 0.0094 across the five fixed seeds. Relative to the same-architecture student trained from scratch (0.9210 ± 0.0029 Dice /0.7726 ± 0.0057 ${BF1}_{t3}$), the full model improves Dev Dice by +0.0090 and Dev ${BF1}_{t3}$ by +0.0282. At the paired-seed level, the Dev ${BF1}_{t3}$ difference has a 95% CI of [+0.0158, +0.0406] and Cohen’s $d_{z}$ of 2.83; the exact two-sided sign-flip *p*-value is 0.0625 with five seed pairs, so we report this as effect-size and interval evidence rather than as a dichotomous *p* < 0.05 claim. Using the same three additional pre-registered seeds (7, 777, 88,888) introduced below for the four-external extension, we recomputed the identical paired statistic for Dev ${BF1}_{t3}$ over all eight seed pairs: 0.8042 ± 0.0085 (full) vs. 0.7724 ± 0.0083 (scratch), paired difference +0.0317, t-based 95% CI [+0.0220, +0.0415], Cohen’s $d_{z}$ = 2.73, exact two-sided sign-flip *p* = 0.0078 (paired *t*-test *p* = 0.000113 as a cross-check)—this is the same exact sign-flip test as the five-seed result, and the smaller *p*-value reflects the doubled paired sample staying eight-for-eight in the same direction rather than a different or more lenient test being substituted; the five-seed headline numbers (0.8008 ± 0.0094/ 0.7726 ± 0.0057) are retained elsewhere in this paper as the primary reported values. The four-external Dice comparison for the same full-vs-scratch pair shows the identical exact *p* = 0.0625 at five seeds (+0.0137, 95% CI [−0.0025, +0.0300]); to test whether this reflects the small paired sample rather than a fragile effect, we re-trained both variants under three additional pre-registered seeds (7, 777, 88,888) and recomputed the same paired statistic over all eight seed pairs: four-external Dice 0.8217 ± 0.0068 (full) vs. 0.8076 ± 0.0089 (scratch), paired difference +0.0141, t-based 95% CI [+0.0032, +0.0251], Cohen’s $d_{z}$ = 1.08, exact two-sided sign-flip *p* = 0.015625—a tighter interval and a smaller exact *p*-value than the five-seed result, without altering the five-seed headline numbers reported elsewhere in this paper. The same student-only efficiency measurements correspond to Table 1 and define the compact-inference target of this study. Measured under this protocol, the model has 3.72M parameters, 1.76 GFLOPs, 140.9 FPS, 7.10 ms latency, and 163.3 MB peak inference memory on a (1, 3, 352, 352) RTX-4090 AMP warmup-30 timed-100 thop [34] protocol; the same student profiled on a second architecture, an edge Jetson Orin Nano, runs at 33.9 FPS under the same protocol (Table 1).

Under the same locked recipe, we ablate the three KD signals—response distillation (A), boundary-probability distillation (B), and detail-feature alignment (C)—across all single, pairwise, and full combinations (Table 2). Boundary-probability distillation has the highest mean Dev ${BF1}_{t3}$ among the single KD signals: response KD reaches 0.7860 ± 0.0062 ${BF1}_{t3}$, boundary KD reaches 0.7990 ± 0.0127, and detail KD alone reaches 0.7742 ± 0.0064, close to scratch. All eight rows in Table 2, including the pairwise rows, are five-seed results under the same frozen teacher. The response+boundary row (A+B) reaches Dev ${BF1}_{t3}$ 0.8009 ± 0.0034, statistically tied with the full A+B+C row at 0.8008 ± 0.0094 (paired diff –0.000063, exact sign-flip *p* = 1.0000); therefore, the detail signal should not be interpreted as improving the Dev boundary endpoint when added to A+B. Instead, the data show a context-dependent effect: detail alone is weak, and although boundary+detail (B+C) gives the highest pairwise Dev ${BF1}_{t3}$ (0.8032 ± 0.0088) its four-external Dice falls to 0.8101, below boundary distillation alone (0.8147)—so the detail signal does not transfer to held-out data on its own and contributes only within the full combination, which reaches the highest mean four-external Dice in this same-architecture KD ablation family (0.8225 ± 0.0071). We report the full A+B+C combination as the pre-specified configuration because all three signals are individually motivated in the loss design, not because it maximizes a held-out score; A+B is a statistically tied, simpler variant on the Dev boundary endpoint. Table 2’s eight rows together give the per-signal contribution and pairwise-interaction decomposition: each of the three single-signal rows isolates one distillation signal’s independent effect, and each pairwise/full row isolates the corresponding interaction.

Against the evaluated compact and mid-light baselines under the same locked recipe (Table 3), BDKD-Net leads on Dev Dice and remains competitive on Dev ${BF1}_{t3}$ on the locked internal dev split. The compact and mid-light row chain on (parameters/Dev ${BF1}_{t3}$/Dev Dice) is BDKD-Net 3.72M 0.8008/0.9300 against MobileNetV2-UNet [35] 3.00M 0.7892/0.8923, LRASPP-MV3-L [36] 3.22M 0.7835/0.9051, MobileNetV3-Large-UNet [36] 4.24M 0.8043/0.9101, and DeepLabV3-MV3-L_pretrained [37] 11.02M 0.7956/0.9104. In the higher-capacity baseline audit (Table 4), Polyp-PVT reaches the highest harmonized four-external Dice (0.8417 ± 0.0091), above BDKD-Net (0.8225 ± 0.0071). BDKD-Net is nevertheless substantially smaller and faster under the fresh efficiency protocol: 3.72M parameters, 1.76 GFLOPs, and 140.9 FPS versus Polyp-PVT’s 25.11M parameters, 10.02 GFLOPs, and 72.0 FPS. This reflects a compact efficiency–accuracy trade-off under the present protocol; the Discussion’s limitations paragraph scopes what these efficiency and accuracy measurements do and do not support. For full BDKD-Net, the per-external Dice/${BF1}_{t3}$ values are CVC-ColonDB 0.7747 ± 0.0068/0.6714 ± 0.0068, CVC-300 0.8716 ± 0.0091/0.8098 ± 0.0156, ETIS 0.8099 ± 0.0113/0.6680 ± 0.0152, and BKAI 0.8339 ± 0.0070/0.7289 ± 0.0172 across the five fixed seeds.

To refresh the comparison window with recent methods, we audited a set of 2024–2026 knowledge-distillation, lightweight, and boundary-focused polyp segmentation methods and added every one for which a faithful evaluation was possible. Three were evaluated from the authors’ official released weights under our unified four-external protocol (sigmoid > 0.5): KDAS [17], a 3.65M PVTv2-B0 distilled student, reaches four-external Dice 0.7956 (Table 3); MEGANet-Res2Net [10] (44.19M, WACV 2024) reaches 0.7979 (Table 4) and DepthPolyp [23] reaches 0.7493 with ${BF1}_{t3}$ 0.6240. We additionally retrained KDAS, MEGANet-Res2Net, and EMCAD [21] under our split with five seeds and each method’s faithful recipe (Table 5), reaching four-external Dice 0.7900 ± 0.0041, 0.7806 ± 0.0044, and 0.7835 ± 0.0093, respectively, the seed-42 KDAS and MEGANet-Res2Net retrainings reproduce their official-weight scores within 0.012 and 0.014. All of these recent 2024–2026 baselines stay below BDKD-Net’s four-external Dice of 0.8225 under matched evaluation. The scratch PVTv2-B0 student already reaches 0.8088 four-external Dice, above all of these recent baselines; because the backbone is held constant across the scratch and full variants, the additional +0.0137 isolates the contribution of the full composite knowledge-distillation objective (response, boundary-probability, and detail-feature signals together), of which the single-signal boundary-probability contribution is +0.0059 four-external Dice over scratch (Table 2), with the response and detail signals and their interactions accounting for the remainder.

The remaining recent methods could not be placed on a comparable quantitative footing and are cited as method references only. LGPS [22] provides public code whose forward pass we verified reproduces the paper’s 1.07M-parameter generator, but its official weights are hosted on login-restricted cloud storage and were not retrievable. LiteBounD [24] provides a public repository that currently contains documentation and figures only, without a runnable model or released weights (checked June 2026), so it cannot be evaluated under our protocol. De-LightSAM [25] releases a multi-modality weight archive that does not include the colonoscopy-specific weights described in the paper, so its reported colonoscopy configuration cannot be evaluated as published. MEGANet-W [26]—a 2025 wavelet-driven variant distinct from the 2024 Laplacian-based MEGANet-Res2Net evaluated above—has no public repository, and its reported scores use a min–max normalization protocol rather than our fixed sigmoid > 0.5 binarization; the size of this protocol gap is visible directly on MEGANet-Res2Net, whose own paper [10] reports min–max Dice of 79.3, 89.9, and 73.9 on CVC-ColonDB, CVC-300, and ETIS, respectively, (their paper does not evaluate BKAI-IGH-NeoPolyp), averaging 0.810 on these three datasets—about 0.019 Dice above our sigmoid > 0.5 re-evaluation on the same three datasets (0.792), not the four-external average (their paper reports no BKAI score to compare against). MEGANet-W’s self-reported numbers are, therefore, not directly comparable to the values in this paper. MEGANet-W is a full-size 416-input model and does not report BKAI, further limiting any like-for-like comparison with our compact student.

Inside the detail-feature alignment pathway, we probe the adapter operator as a design choice by comparing three adapter variants—PartialConv3 [31], a standard 3 × 3 convolution, and a no-detail-adapter control—on Dev Dice, Dev ${BF1}_{t3}$, and GFLOPs under the same recipe, across four seeds (Table 6). The three rows on (Dev Dice/Dev ${BF1}_{t3}$/GFLOPs) are full_bdkd_partialconv3 0.9300 ± 0.0034/0.8041 ± 0.0057/1.76; bdkd_no_detail_adapter 0.9283 ± 0.0033/0.7981 ± 0.0143/1.72; bdkd_standard_conv3 0.9281 ± 0.0020/0.7998 ± 0.0081/ 2.01. This four-seed set is distinct from the five-seed full-model headline in Table 2 (Dev ${BF1}_{t3}$ 0.8008 ± 0.0094), so the two are not directly comparable. Against the standard 3 × 3 convolution variant, PartialConv3 has lower GFLOPs and a paired-seed advantage that reaches significance on Dev MAE (p=0.035, Cohen’s d=−1.15, large effect; Dice/${BF1}_{t3}$/IoU deltas are directionally consistent but not significant at n=4) and a substantially larger external-generalization gap (no-EAD four-external average +1.29pp). We, therefore, treat PartialConv3 as a supported design choice for the detail-feature adapter rather than a general hardware-deployment conclusion, on the basis of this four-seed paired comparison.

**Figure 3:**
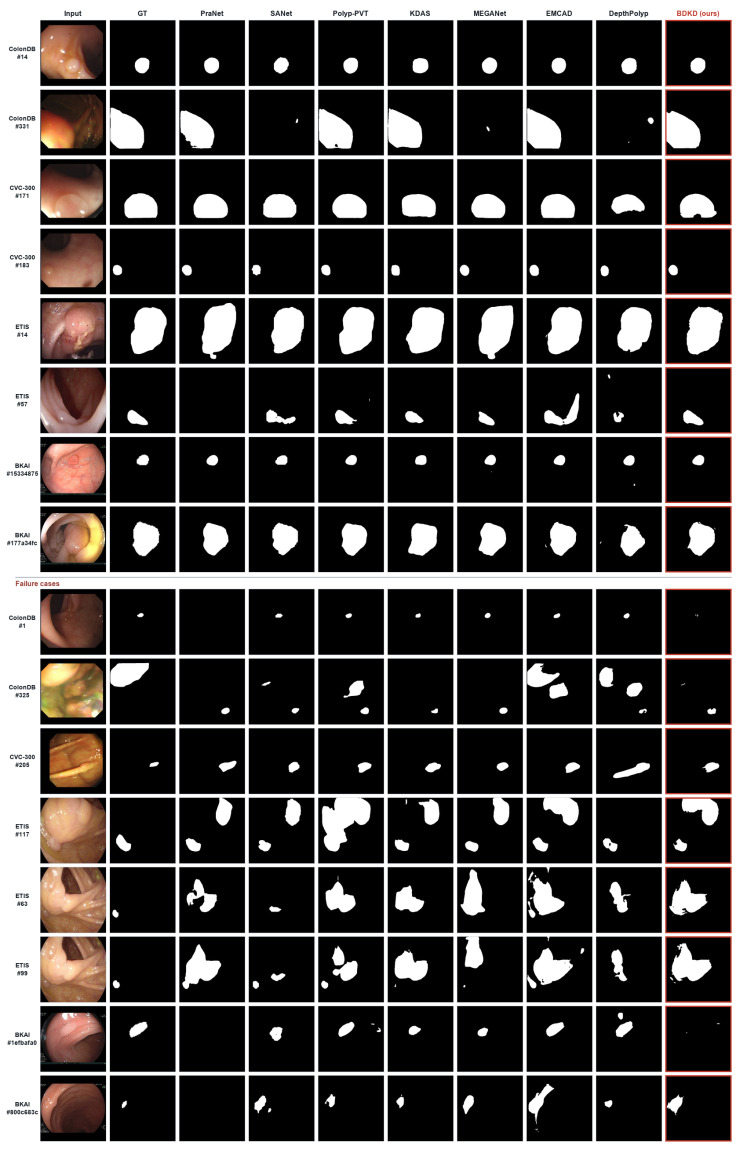
Qualitative Comparison of the external polyp benchmarks. Each row is one case; the top block shows representative cases (two per dataset) from CVC-ColonDB, CVC-300, ETIS, and BKAI-IGH-NeoPolyp, and the bottom block shows eight failure cases selected from the ranked external pool by BDKD-Net Dice (two ColonDB, one CVC-300, three ETIS, two BKAI; not evenly split across datasets). Columns are the input image, the ground-truth mask, the predicted masks of seven baselines (PraNet, SANet, Polyp-PVT, KDAS, MEGANet [10] (i.e., MEGANet-Res2Net), EMCAD, DepthPolyp), and BDKD (ours, red). All predictions use each method’s own checkpoint under the unified sigmoid > 0.5 binarization, the same checkpoints and protocol as the quantitative tables; an all-black panel denotes an empty (all-background) prediction.

We further tested whether the headline result depends on a fragile protocol choice. A local loss-weight sweep around the main response/boundary/detail weights of 0.5/1.0/0.25 shows that the main setting was the highest observed setting in this limited local sweep, with four perturbations within –0.0062 four-external Dice of the main result (Table 7). A training-band-width sweep gives band1, band3, and band5 four-external Dice values of 0.8227, 0.8225, and 0.8269, respectively. Because the band5 increase over the locked band3 protocol is +0.0044 and remains within the main seed standard deviation (0.007), we report it as sensitivity evidence rather than replacing the pre-declared main protocol, which was registered as band3 before this sweep was run. A teacher-mask-threshold sweep (θ∈{0.3,0.4,0.6,0.7}, 3 seeds each) gives four-external Dice 0.8189, 0.8238, 0.8126, and 0.8233, respectively, against the locked θ=0.5 main protocol at 0.8225 (Table 7); the largest deviation (θ=0.6, −0.0099) is within that setting’s own seed standard deviation (±0.0115), so we do not find evidence that 0.5 is a fragile or arbitrary choice within this local range, and retain it as the pre-registered main protocol. The evaluation-side BF1 tolerance audit is also monotonic across 1, 3, and 5 pixels; for example, ETIS BF1 increases from about 0.51 to 0.67 to 0.73 across those tolerances, so ${BF1}_{t3}$ is treated as a mid-point computer-vision boundary endpoint, not a clinical millimetre measurement. Finally, a 3-seed dual-checkpoint sensitivity family shows that selecting by best Dev ${BF1}_{t3}$ instead of best Dev Dice would raise BDKD-full four-external Dice by +0.010 within run, but the main paper keeps the locked best-Dev-Dice checkpoint-selection rule to avoid post-hoc endpoint selection. A qualitative comparison against seven baselines on representative external and failure cases is shown in Figure 3, using each method’s own checkpoint under the unified protocol.

## 4. Discussion

The main finding of this study is that boundary-probability knowledge distillation acts as the task-adapted contributor on a 3.72M compact polyp segmentation student, lifting the boundary F1 at 3-pixel tolerance on the locked internal dev split from 0.7726 ± 0.0057 (scratch) to 0.7990 ± 0.0127 as a single boundary-KD signal and to 0.8008 ± 0.0094 under the full combination. On the same dev split, Dev Dice rises from 0.9210 ± 0.0029 (scratch) to 0.9300 ± 0.0029 under the full combination. Under student-only inference, the full combination measures 3.72M parameters, 1.76 GFLOPs, and 140.9 FPS on the (1, 3, 352, 352) RTX-4090 AMP protocol: the boundary-probability signal, therefore, carries the boundary-sensitive gain at no additional inference cost relative to the scratch student.

The two endpoints respond asymmetrically to boundary supervision. Boundary KD alone lifts Dev ${BF1}_{t3}$ to 0.7990 ± 0.0127, whereas response KD alone reaches 0.7860 ± 0.0062 and detail KD alone remains close to scratch at 0.7742 ± 0.0064. The pairwise rows show that the detail-feature signal is context-dependent rather than monotonic: A+B is statistically tied with full A+B+C on Dev ${BF1}_{t3}$ (0.8009 ± 0.0034 vs. 0.8008 ± 0.0094), while B+C gives the highest pairwise Dev ${BF1}_{t3}$ (0.8032 ± 0.0088) but does not carry that lead to held-out data (four-external 0.8101, below boundary distillation alone), so the full model’s strongest observed evidence is the best four-external Dice within the same-architecture KD ablation family rather than the highest Dev ${BF1}_{t3}$. Together with Table 2, this is the per-signal contribution and pairwise-interaction decomposition for the KD signals: each single-signal row isolates one distillation signal’s independent effect, and each pairwise or full row isolates the corresponding interaction between signals. To formalize the interaction claim, we compute the standard $2^{3}$ factorial contrast (Yates convention, effect estimate = contrast/4) for all three pairwise interactions and the three-way interaction on Dev ${BF1}_{t3}$, using the same five paired seeds as Table 2: the response × boundary interaction’s 95% CI excludes zero and is negative (mean −0.0062, 95% CI [−0.0097,−0.0027], 5/5 seeds same sign, exact sign-flip p=0.0625, paired *t*-test p=0.0078), which quantifies the sub-additivity between the two signals underlying the A+B/A+B+C tie reported above; the response × detail and boundary × detail interactions and the three-way interaction do not show this pattern at this seed count (all 95% CIs contain zero).

Among existing polyp knowledge-distillation routes, the load-bearing supervision signal of BDKD-Net—boundary-probability distillation on the morphology-dilated teacher boundary band—is a distinct supervision class from the routes pursued by KDAS, PRISM, and MicroAUNet. KDAS [17] transfers attention maps from teacher to student through symmetric guidance, so the distillation target is the spatial attention map at intermediate stages, without an explicit probability-band restriction on the prediction maps. PRISM [18] drops the separate teacher network altogether and builds a momentum teacher from an exponential moving average of the student’s own weights, then minimizes a sigmoid-scaled mean squared error between teacher and student predictions over the full image, without a boundary mask or a compact-student target. MicroAUNet [19] trains a 0.025M student through a two-stage curriculum: stage one imitates the teacher’s hidden features with an L2 mimicry loss combined with KL divergence on softened semantic predictions; stage two adds an output-level contrastive loss on positive and negative pairs partitioned by teacher confidence; neither stage applies a prediction-level binary cross-entropy restricted to a teacher boundary band. Set against this neighbor set, BDKD-Net’s load-bearing supervision class is the band-restricted prediction-level binary cross-entropy on the morphology-dilated teacher mask, which carries the boundary-endpoint lift on the locked dev split under the stated efficiency protocol.

Several scope limitations bound the present findings, and we flag here explicitly that cross-method comparisons throughout this paper are not all under identical evaluation protocols or dataset configurations: MEGANet-Res2Net’s officially reported score uses a min–max normalization protocol rather than our fixed sigmoid>0.5 binarization, with the resulting protocol gap (∼0.019 Dice on the three datasets their paper reports) quantified directly in the Results baseline-audit discussion above, KDAS’s Dev-split columns are in-domain rather than held-out because its training pool overlaps our internal dev split (Table 3 footnote), and CFANet, M2SNet, and SSFormer-L carry reproduction-fidelity or aggregate-only caveats (Table 4 footnote); readers should treat cross-row comparisons in Table 3 and Table 4 as protocol-aware rather than strictly like-for-like. In addition, full-size baselines remain stronger on Dev Dice, the external comparison is restricted to the four external public benchmarks, the efficiency profile is tied to fixed per-device measurement protocols, the static boundary supervision inherits the teacher’s boundary prior, and the loss weights are held fixed across ablation variants. In this harmonized baseline audit, BDKD-Net’s four-external average reaches Dice 0.8225, below the larger Polyp-PVT row at 0.8417 (and the context-only SSFormer-L row at 0.8264) but above the official-weight DepthPolyp result of 0.7493. The efficiency profile of 3.72M parameters, 1.76 GFLOPs, 140.9 FPS, 7.10 ms latency, and 163.3 MB peak inference memory is measured under (1, 3, 352, 352) RTX-4090 AMP; on a second architecture, an edge Jetson Orin Nano (MAXN_SUPER, JetPack/L4T R36.5), the same student runs at 33.9 FPS, 29.48 ms latency, and 41.3 MB peak memory under the same protocol with identical 3.72M parameters and 1.76 GFLOPs (Table 1). These per-device numbers are not directly comparable to each other or to literature values measured under different hardware or protocols, and are reported only as efficiency measurements. The boundary band is derived from the 0.5-thresholded teacher mask; to test whether this static teacher-derived prior biases generalization, we re-trained the full model with the band instead derived from the ground-truth mask, holding everything else fixed across the same five seeds. The teacher-derived and ground-truth bands are statistically indistinguishable on every endpoint (paired Δ = ground-truth − teacher: Dev Dice −0.0010, 95% CI [−0.0040,+0.0021]; Dev ${BF1}_{t3}$ −0.0049, [−0.0207,+0.0108]; four-external Dice −0.0099, [−0.0243,+0.0045]; all CIs contain zero, n=5), so the static teacher-derived band does not measurably harm generalization relative to a ground-truth band and is directionally on par with it, although the four-external comparison is underpowered at five seeds (Cohen’s $d_{z}=-0.86$, exact p=0.125). The response, boundary, and detail-feature weights remain held at 0.5, 1.0, and 0.25 across ablation variants, with no per-variant joint retuning. The sensitivity sweep supports local stability but does not establish a globally optimal hyperparameter setting; the marginally higher band5 result is reported without replacing the locked band3 main protocol, which was pre-registered before this sensitivity sweep was run. ${BF1}_{t3}$ is a computer-vision boundary endpoint; clinical millimetre-scale interpretation would require calibrated endoscope distance and magnification information and is outside this study. The four external benchmarks (CVC-ColonDB, CVC-300, ETIS, and BKAI-IGH-NeoPolyp) are acquired at different institutions and are never seen during training, tuning, or checkpoint selection, so the four-external average already serves as a cross-dataset, cross-centre generalization estimate; leave-one-dataset-out (LODO) robustness and explicit domain-adaptation metrics remain future work. Forward-looking work includes multi-center prospective validation, a theoretical analysis of band-restricted distillation, dynamic-teacher variants, and multi-task transfer to other low-contrast medical-image segmentation tasks such as skin lesion or endoscopic ulcer segmentation; prospective clinical evaluation lies outside this scope.

## 5. Conclusions

Boundary-probability knowledge distillation, supervising the student probability map on the morphology-dilated boundary band of the teacher’s σ-thresholded mask, has the highest mean Dev ${BF1}_{t3}$ among the single KD signals on a 3.72M compact polyp segmentation student, reaching Dev ${BF1}_{t3}$ 0.7990 ± 0.0127 compared with 0.7726 ± 0.0057 for scratch and 0.7860 ± 0.0062 for response distillation alone. The full BDKD-Net reaches Dev Dice 0.9300 ± 0.0029, Dev ${BF1}_{t3}$ 0.8008 ± 0.0094, and four-external Dice 0.8225 ± 0.0071, while holding the inference budget at 1.76 GFLOPs and 140.9 FPS. The auxiliary detail-feature alignment plays a context-dependent supporting role: detail alone is weak, B+C is the highest pairwise Dev ${BF1}_{t3}$ row, and full A+B+C has the highest mean four-external Dice within the same-architecture KD ablation family. In the broader baseline audit, the larger Polyp-PVT row (and the context-only SSFormer-L row) report higher four-external Dice, so BDKD-Net is best understood as a compact efficiency–accuracy operating point under the evaluated protocol. The Discussion outlines the corresponding scope limitations and forward-looking work.

## Figures and Tables

**Table 1:** Inference-time efficiency profile of the BDKD-Net student inference graph (teacher dropped after training), measured on two architectures: a server GPU (RTX 4090) and an edge device (Jetson Orin Nano, MAXN_SUPER, JetPack/L4T R36.5). All values use the protocol input (1, 3, 352, 352) with AMP, 30 warmup and 100 timed iterations; GFLOPs are the thop multiply-add count. Because the PVTv2-B0 backbone’s attention blocks execute through PyTorch 2.1.0’s fused scaled-dot-product-attention kernel, thop’s hook-based profiler—which only attributes compute to registered nn.Module instances—does not count the attention query-key and attention-value matrix multiplications; a controlled toggle between the fused and unfused attention code paths confirms this (identical thop totals either way), and a configuration-derived manual estimate of the omitted attention matmuls is 0.23 GFLOPs, so the reported 1.76 GFLOPs excludes this term and the architecture’s full compute is approximately 1.99 GFLOPs; this is a general limitation of thop for any transformer-attention model, applies identically to the Polyp-PVT row reported below, and does not affect the convolutional baselines. Parameters and GFLOPs are hardware-independent and identical on both devices; FPS, latency, and peak memory are per-device under each device’s runtime, so the two columns are not directly comparable to each other or to literature values measured under different settings. The RTX-4090 column was re-profiled under GPU driver 595.58.03 (22 June 2026). These are efficiency measurements, not deployment-readiness claims.

Metric	RTX 4090	Jetson Orin Nano
Parameters	3.72M	3.72M
GFLOPs	1.76	1.76
FPS	140.9	33.9
Latency per image	7.10 ms	29.48 ms
Peak inference memory	163.3 MB	41.3 MB

**Table 2:** KD ablation on the locked Kvasir-SEG + CVC-ClinicDB internal dev split (161 images). All variants share the 3.72M PVTv2-B0 + decoder64 student architecture and the same training recipe; only the loss configuration varies. All rows are five-seed means  ±  standard deviations under the same frozen teacher and fixed seeds (42, 1888, 2024, 9999, 31,415). The four external columns averages CVC-ColonDB, CVC-300, ETIS, and BKAI.

Variant	Dev Dice	Dev BF1_*t*3_	Four-Ext. Dice
Scratch (no KD)	0.9210 ± 0.0029	0.7726 ± 0.0057	0.8088 ± 0.0076
+Response (A)	0.9264 ± 0.0035	0.7860 ± 0.0062	0.8093 ± 0.0068
+Boundary (B)	0.9290 ± 0.0047	0.7990 ± 0.0127	0.8147 ± 0.0092
+Detail (C)	0.9228 ± 0.0020	0.7742 ± 0.0064	0.8113 ± 0.0050
A+B	0.9300 ± 0.0021	0.8009 ± 0.0034	0.8190 ± 0.0041
A+C	0.9268 ± 0.0019	0.7853 ± 0.0121	0.8148 ± 0.0072
B+C	**0.9316 ± 0.0033**	**0.8032 ± 0.0088**	0.8101 ± 0.0083
**Full A+B+C**	0.9300 ± 0.0029	0.8008 ± 0.0094	**0.8225 ± 0.0071**

Boldface marks the best value in each metric column; the full A+B+C row label is bolded to identify the pre-specified full BDKD-Net configuration.

**Table 3:** Compact and mid-light baseline comparison on the locked internal dev split, under the same training recipe and 352 × 352 input. BDKD-Net (3.72M) is the proposed student; other rows are MobileNet/LRASPP/DeepLabV3 backbones evaluated under an identical recipe. Dev BF1_*t*3_ is the boundary-fidelity endpoint; four-ext. Dice is the four-external polyp benchmark average. Except for BDKD-Net, Table 3 entries are single-run protocol values unless repeated-run statistics are explicitly shown.

Method	Params (M)	GFLOPs	FPS	Dev Dice	Dev BF1_*t*3_	Four-Ext. Dice
MV2-UNet [35] ^‡^	3.00	2.99	221.8	0.8923	0.7892	0.6990
LRASPP-MV3 [36]	3.22	0.98	187.3	0.9051	0.7835	0.7181 ± 0.0048
MV3-UNet [36]	4.24	4.55	196.7	0.9101	0.8043	0.7214 ± 0.0065
DeepLabV3-MV3 ∗ [37]	11.02	4.71	170.3	0.9104	0.7956	0.7226 ± 0.0052
KDAS ^†^ [17]	3.65	1.69	119.3	n/a	n/a	0.7956
**BDKD-Net (ours)**	**3.72**	**1.76**	140.9	**0.9300 ± 0.0029**	**0.8008 ± 0.0094**	**0.8225 ± 0.0071**

Boldface marks the proposed BDKD-Net row and its reported values. Params/GFLOPs/FPS use the fresh RTX-4090 AMP warmup-30 timed-100 profiling bundle (driver 595.58.03, 22 June 2026 refresh; all efficiency rows in this paper re-profiled together so FPS values are mutually comparable). n/a indicates that no reliable comparable value was available for this entry (the four-external evaluation did not complete, or the entry is in-domain rather than held-out), and the value is, therefore, not imputed. ∗ DeepLabV3-MV3: pretrained variant; the scratch variant is internal-only (non-converged) per the project registry. ^†^ KDAS: official-weight single-run external evaluation of the authors’ released 3.65M PVTv2-B0 distilled checkpoint, re-scored under our four-external protocol (sigmoid > 0.5); its efficiency is profiled under the same bundle, but because the authors’ training pool overlaps the internal dev split, the Dev columns are in-domain and reported as n/a, leaving the four-external average as the only comparable cross-method accuracy value. A five-seed harmonized retraining of KDAS under our split is reported in Table 5. ^‡^ MV2-UNet: an earlier from-scratch reproduction reached external Dice 0.307 ± 0.008, well below every other row and traced to the MobileNetV2 encoder lacking ImageNet-pretrained initialization (unlike the pretrained-encoder rows in this table); this row now reports a repaired single-run (seed 42) reproduction with the encoder initialized from torchvision’s native ImageNet-1k MobileNetV2 weights, holding the decoder untrained as before, which recovers a normal dev-to-external generalization gap (0.8923 Dev Dice to 0.6990 four-external Dice, comparable to the other pretrained-encoder compact rows) and confirms the missing pretrained initialization as the root cause; the four-external Dice of 0.6990 remains modestly below the MobileNetV3-based compact rows, consistent with a somewhat weaker encoder paired with a deliberately lightweight (∼2.7M) decoder.

**Table 4:** Higher-capacity baseline comparison with four-external audit tracks and fresh RTX-4090 efficiency profiling where available. The four-ext. Dice column is the four-external polyp benchmark average used for the external comparison in this paper; rows with incomplete or context-only evidence are marked in the note rather than imputed.

Method	Params (M)	GFLOPs	FPS	Dev Dice	Four-Ext. Dice
PraNet [6]	32.55	13.15	61.6	0.9761	0.8182 ± 0.0208
SANet [7]	23.90	11.34	103.0	0.9489	0.7872 ± 0.0236
Polyp-PVT [8] ^‖^	25.11	10.02	72.0	**0.9830**	**0.8417 ± 0.0091**
CFANet [9] ^§^	25.83	55.36	59.5	0.9697	0.7015 ± 0.0100
M2SNet [38] ^†^	29.88	33.29	55.7	0.9702	n/a
SSFormer-L [39] ^‡^	66.22	n/a	n/a	n/a	0.8264
MEGANet-Res2Net [10] ^¶^	44.19	28.85	48.2	0.9767	0.7979
**BDKD-Net (ours)** ^‖^	**3.72**	**1.76**	**140.9**	0.9300	0.8225 ± 0.0071

Boldface marks the best accuracy values in the table and the proposed BDKD-Net row/efficiency values where visually emphasized. n/a indicates that official weights, prediction maps, compatible aggregate metrics, or fresh profiling were unavailable for this entry; it does not indicate a zero value. All reported FPS values use the same RTX-4090 driver-595.58.03 (22 June 2026) profiling bundle as Table 1, so they are mutually comparable. Dev Dice values are retained from the internal-dev evaluation, while four-external values use the harmonized evaluation track when available. ^§^ CFANet is included as a caveated harmonized reproduction: the edge target is generated from masks; the underlying 200-epoch training loop does not evaluate on the dev split during training and saves only a single last-epoch checkpoint, so best-Dev-Dice checkpoint selection was not applied and no intermediate checkpoint remains available to apply it retrospectively. ^†^ M2SNet: aggregate-only accuracy result with partial weight load caveat; FPS (55.7) is from a separate fresh RTX-4090 inference-only profiling pass with a strict, complete weight load (params 29.74M, within rounding of the reported 29.88M), so the FPS figure is not subject to the same partial-load caveat as the accuracy row; the reported GFLOPs (33.29) is measured with fvcore.FlopCountAnalysis rather than thop, because thop’s hook-based counting misses this architecture’s functional (non-nn.Module) convolution and undercounts a convolution submodule that is invoked 20 times within a single forward pass (hooks on a reused module instance record only the last call), together accounting for the previously reported, now superseded 17.06 value; fvcore’s trace-based counting captures both cases and reproduces the same GFLOPs via an independent manual per-call accounting of the affected submodule. ^‡^ SSFormer-L: supplementary aggregate-prior-mmseg result without prediction maps; reported for context only. A best-effort attempt to load the released weights under the current MMSeg version found an incompatible backbone key naming (patch_embed1-4/block1-4 vs. the current layers.0-3); a hand-built PyTorch reimplementation matching the released weights’ key naming loaded with zero missing or unexpected keys but produced degenerate, near-uniform predictions (Dice ≈ 0 on held-out data), indicating its decode head does not correctly reconstruct the official segmentation head despite the matching key names, so a matched key namespace alone is not a sufficient fix. GFLOPs/FPS/Dev-Dice, therefore, remain n/a rather than a validated fresh reproduction. ^¶^ MEGANet-Res2Net [10] (WACV 2024; Res2Net-50 backbone): official-weight single-run four-external evaluation under our protocol (sigmoid > 0.5), so no seed standard deviation is reported; its five-seed harmonized retraining under our split appears in Table 5, and its GFLOPs are counted with GPU-side thop because the released forward pass hard-codes device placement. This 2024 Laplacian edge-guided method is distinct from MEGANet-W [26] (wavelet-based), which is cited as a method reference only. ^‖^ Polyp-PVT and BDKD-Net: both are PVTv2-based transformer students whose attention blocks execute through PyTorch’s fused scaled-dot-product-attention kernel; thop’s hook-based profiler does not attribute compute to this functional call (confirmed by toggling the fused/unfused attention code path, which leaves the thop total unchanged), so the reported GFLOPs for both rows exclude the attention query-key and attention-value matrix multiplications. This is a general thop limitation for transformer-attention models, not specific to either method; it does not affect the convolutional baselines in this table. For BDKD-Net, a configuration-derived manual estimate of the omitted attention compute is 0.23 GFLOPs (full architecture ≈1.99 GFLOPs including attention); see Table 1 for the derivation. The reported 1.76/10.02 values are retained for within-table comparability under the shared thop protocol.

**Table 5:** Supplementary five-seed harmonized retraining of recent knowledge-distillation and efficient-decoder baselines under our unified split (pooled Kvasir-SEG + CVC-ClinicDB, 10% internal dev at seed 42), five seeds {42, 1888, 2024, 9999, 31,415}, with each method trained under its own faithful recipe and selected by the best internal-dev Dice. The four-external column is the five-seed mean ± standard deviation over CVC-ColonDB, CVC-300, ETIS, and BKAI; Dev Dice is the five-seed mean on the held-out internal-dev split. Params/GFLOPs/FPS are the architecture-level efficiency from the same RTX-4090 driver-595.58.03 profiling bundle as Table 1. This harmonized-retraining track complements the official-weight single-run KDAS and MEGANet-Res2Net rows in Table 3 and Table 4.

Method (Retrained Under Our Split)	Params (M)	GFLOPs	FPS	Four-Ext. Dice	Dev Dice
KDAS [17]	3.65	1.69	119.3	0.7900 ± 0.0041	0.9125
MEGANet-Res2Net [10]	44.19	28.85	48.2	0.7806 ± 0.0044	0.9231
EMCAD [21]	3.92	1.59	91.1	0.7835 ± 0.0093	0.9383
**BDKD-Net (ours)**	**3.72**	**1.76**	**140.9**	**0.8225 ± 0.0071**	0.9300

Boldface marks the proposed BDKD-Net row and its reported values. Every model in this table is trained only on our 1451-image training split, so Dev Dice here is a held-out internal-dev value. For KDAS and MEGANet-Res2Net, a seed-42 reproduction check matched the official-weight four-external score within 0.012 and 0.014 (inside the ±0.05 tolerance), confirming the faithful recipe; the five-seed means tabulated here are the harmonized-track values. Params/GFLOPs/FPS are architecture-level and identical to the shared-architecture rows in Table 3 and Table 4 (MEGANet-Res2Net GFLOPs via GPU-side thop). EMCAD has no released polyp-domain weights, so it appears only in this retraining track; at 3.92M parameters and 91.1 FPS it is the closest efficiency neighbour to BDKD-Net but reaches a lower four-external Dice. All retrained baselines remain below BDKD-Net on the four-external average, while the larger Polyp-PVT row in Table 4 (0.8417 ± 0.0091) still exceeds BDKD-Net.

**Table 6:** PartialConv3 adapter ablation inside the detail-feature alignment pathway, framed as a design-choice observation per the ledger C007 boundary. The three variants share the response + boundary + detail loss configuration; only the detail adapter operator varies. Numbers are four-seed mean ± std from a dedicated adapter sweep (distinct seed set from the headline five-seed KD ablation sweep in Table 2, hence not directly comparable to it). A paired *t*-test across the four seeds shows PartialConv3 vs. standard 3 × 3 conv reaches significance on Dev MAE (Δ=−0.0008, p=0.035, Cohen’s d=−1.15, large effect); Dev Dice, Dev BF1_*t*3_, and IoU differences are directionally consistent but not significant at this seed count (p>0.1). Against standard 3 × 3 conv, PartialConv3 also reaches higher external no-EAD Dice (0.8191 vs. 0.8062, +1.29pp; ColonDB +1.15pp, ETIS +1.99pp, BKAI +2.28pp) at 14% fewer GFLOPs (1.76 vs. 2.01) and comparable FPS (130.4 vs. 131.7).

Variant	Dev Dice	Dev BF1_*t*3_	GFLOPs
**Full BDKD + PartialConv3**	**0.9300 ± 0.0034**	**0.8041 ± 0.0057**	**1.76**
BDKD without detail adapter	0.9283 ± 0.0033	0.7981 ± 0.0143	1.72
BDKD with standard 3 × 3 conv	0.9281 ± 0.0020	0.7998 ± 0.0081	2.01

Boldface marks the PartialConv3 configuration used in BDKD-Net and its reported values.

**Table 7:** Protocol-sensitivity checks for the loss-weight, boundary-band-width, and teacher-mask-threshold hyperparameters. The metric is the four-external Dice average over CVC-ColonDB, CVC-300, ETIS, and BKAI. The main protocol (0.5/1.0/0.25 loss weights, training bandwidth 3, teacher-mask threshold θ=0.5, best-Dev-Dice checkpoint selection) was pre-registered before this sweep was run; sweep results including any nominally higher off-main setting are reported without substituting the pre-registered main protocol post hoc.

Check	Setting	Four-Ext. Dice	Δ vs. Main
Loss weights	main 0.5/1.0/0.25	**0.8225**	0.0000
Loss weights	low response 0.25/1.0/0.25	0.8219	−0.0006
Loss weights	low boundary 0.5/0.5/0.25	0.8206	−0.0019
Loss weights	high boundary 0.5/1.5/0.25	0.8170	−0.0055
Loss weights	high detail 0.5/1.0/0.5	0.8164	−0.0061
Band width	band1	0.8227	+0.0002
Band width	band3 (main)	0.8225	0.0000
Band width	band5	0.8269	+0.0044
Teacher threshold	θ=0.3	0.8189	−0.0037
Teacher threshold	θ=0.4	0.8238	+0.0013
Teacher threshold	θ=0.5 (main)	0.8225	0.0000
Teacher threshold	θ=0.6	0.8126	−0.0099
Teacher threshold	θ=0.7	0.8233	+0.0008

Boldface marks the pre-registered main protocol row.

## Data Availability

The data presented in this study are openly available in Kvasir-SEG [2] (https://datasets.simula.no/kvasir-seg/, accessed on 5 July 2026), CVC-ClinicDB/CVC-EndoSceneStill [3], CVC-ColonDB [29], CVC-300, and ETIS-LaribPolypDB [30], with the four external test sets distributed through the PraNet benchmark (https://github.com/DengPingFan/PraNet, accessed on 5 July 2026), and BKAI-IGH-NeoPolyp (Kaggle competition, https://www.kaggle.com/c/bkai-igh-neopolyp, accessed on 5 July 2026). The exact split files, the five fixed seeds, evaluation scripts, and trained checkpoints supporting this study are available from the corresponding author upon reasonable request. Dataset license and access terms should be checked at the source dataset pages listed above.

## Abbreviations

The following abbreviations are used in this manuscript:

$z_{s},z_{t}$	Student and teacher logits.
σ(·)	Sigmoid function.
*y*	Ground-truth binary mask.
$M_{t}$	Teacher hard mask derived from $\sigma (z_{t})$.
*B*	Teacher-derived boundary band used for boundary probability distillation.
$f_{s},f_{t}$	Student and teacher decoder feature maps.
π	1×1 projection from student to teacher feature channels.
*w*	Boundary-band structuring-element width; main protocol uses w=3 pixels.
θ	Teacher-mask threshold; main protocol uses θ=0.5.
